# Malrotation of the Gut in Adults: An Often Forgotten Entity

**DOI:** 10.7759/cureus.2313

**Published:** 2018-03-12

**Authors:** Sushant Bhatia, Sudhir Jain, Chandra B Singh, Lovenish Bains, Rohit Kaushik, Nishant S Gowda

**Affiliations:** 1 General Surgery, Maulana Azad Medical College, New Delhi, India; 2 Maulana Azad Medical College, Lok Nayak Hospital

**Keywords:** malrotation, adults, obstruction, ladd’s procedure, whirlpool sign, corkscrew sign

## Abstract

Malrotation of the gut is a common paediatric condition that usually presents in the first month of life. However, presentation in adults is rare, and as a diagnostic dilemma quite often surprises the surgeon intraoperatively. If this condition is not timely recognized, it may result in disastrous consequences, such as gangrene of the small gut. We present the case of a 21-year-old male who presented to the emergency room with recurrent episodes of colicky abdominal pain and bilious vomiting. Contrast-enhanced computerized tomography (CT) revealed malrotation of the gut. The patient was planned for Ladd’s procedure. Malrotation in adults may present in an acute way due to midgut volvulus or may have a chronic indolent course with recurrent vomiting and abdominal pain. In patients with acute obstruction, this differential should be kept in mind, especially if the patient has no previous abdominal surgery or evidence of tuberculosis. Contrast-enhanced CT is the investigation of choice and reveals typical findings, like whirlpool sign, corkscrew sign, or reversed relation of superior mesenteric artery and vein. The treatment is surgical as failure to do so may result in intestinal gangrene. The procedure of choice is Ladd’s procedure. Every patient, even if asymptomatic, warrants this procedure as it is impossible to predict the timing of catastrophic complications.

## Introduction

Intestinal malrotation is a clinical entity that encompasses partial to complete failure of the 270 degrees’ counterclockwise rotation of the midgut around the superior mesenteric vessels in the fetal life [1]. Its incidence is one in every 200 - 500 newborns [2]. The incidence of symptomatic cases is one in 6,000 newborns [2]. Presentation in adults is very rare. A high index of suspicion is often necessary to diagnose this condition in adults.

## Case presentation

A 21-year-old male presented to the emergency room with complaints of central abdominal pain for the past eight to nine hours and multiple episodes of vomiting. The pain was cramping, located in the central abdominal area. The pain occurred every one to two hours and each episode lasted 15-20 minutes. The pain was associated with episodes of bilious vomiting that provided partial relief. His last bowel movement was two days ago. The patient had been experiencing multiple such episodes for the past two years. No history suggestive of tuberculosis was present. The patient had no previous abdominal surgery. The patient’s blood pressure was 114/82 mm of Hg. The patient’s pulse rate was 92 beats per minute. The systemic examination was unremarkable. The upper abdomen was distended. The abdomen was soft and no tenderness was present. No free fluid was present. Exaggerated bowel sounds were heard. Rectal examination was normal. Abdominal x-rays revealed dilated jejunal loops with abnormal air-fluid levels and no air in the colon.

**Figure 1:**
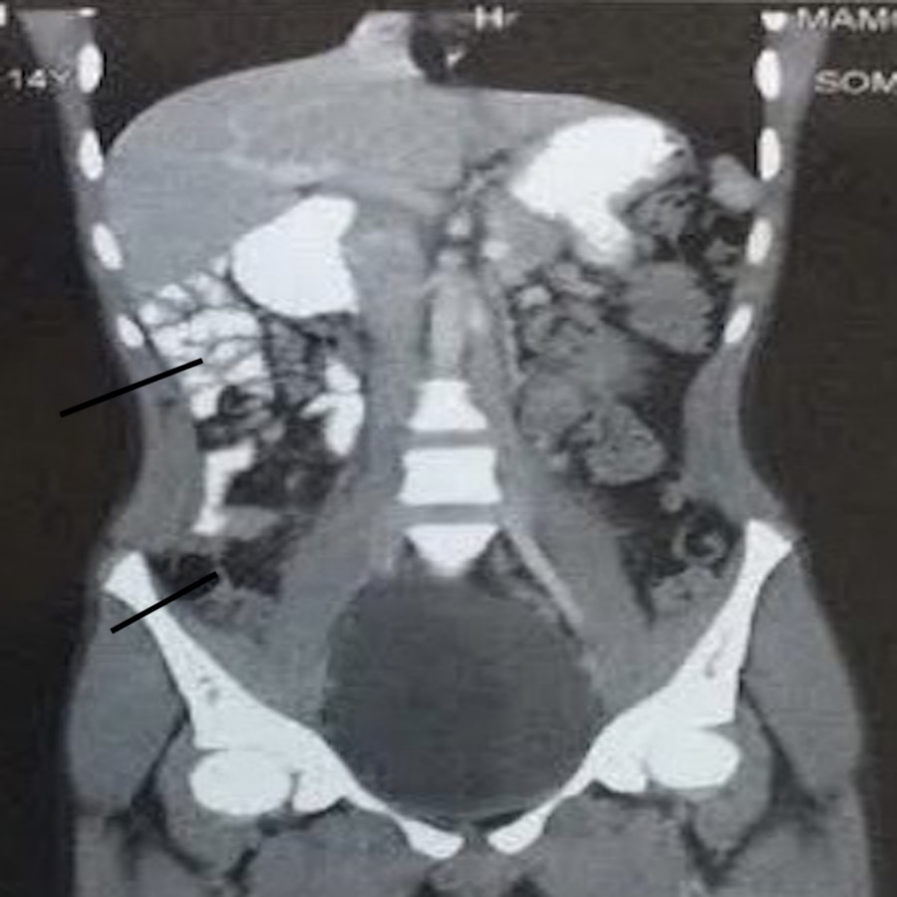
CT showing small bowel loops on the right side and lack of visualization of the caecum in the right iliac fossa CT: computed tomography

**Figure 2:**
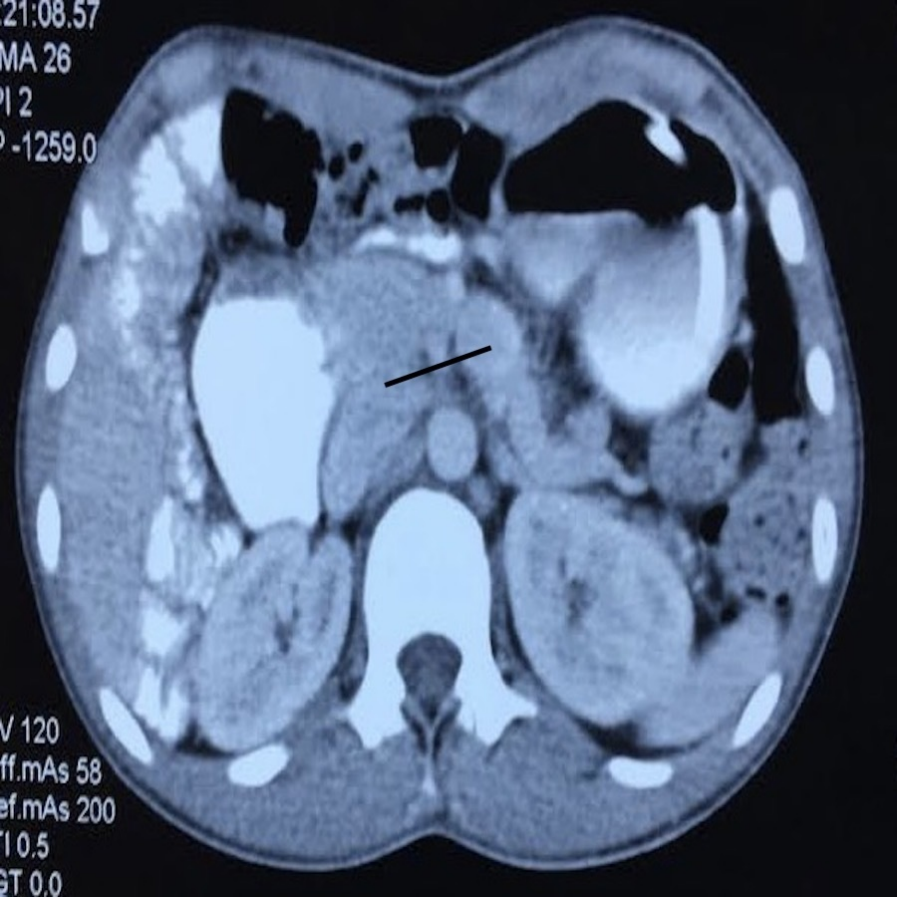
CT showing the superior mesenteric vein on the left CR: computed tomography

**Figure 3:**
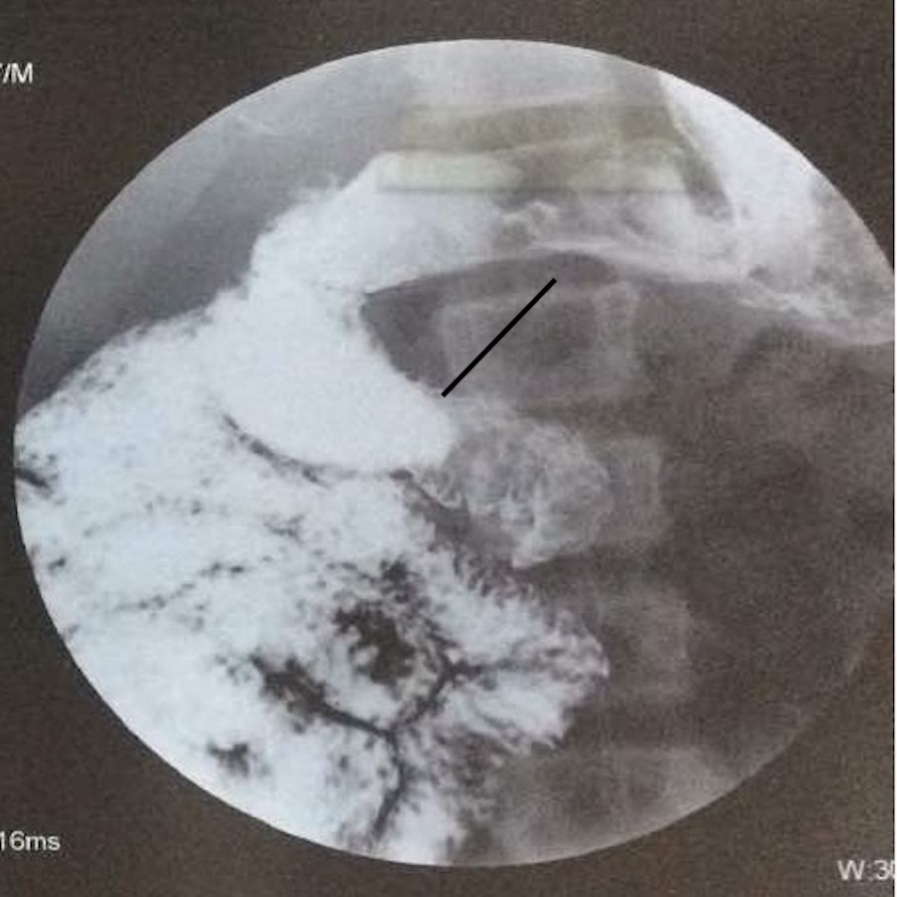
Barium study showing the DJ flexure not crossing to the left side DJ: duodenojejunal

The patient was admitted with a provisional diagnosis of acute intestinal obstruction and was managed with nasogastric decompression and fluids. The pain resolved the next day as did the distension. Patient moved bowels subsequently. The nasogastric output was 800 - 1,000 cc in 24 hours with bilious contents. The patient was allowed liquids gradually and started tolerating semisolids well. Urgent contrast-enhanced computed tomography (CECT) abdomen was done and revealed the duodenojejunal (DJ) flexure to be lying on the right side and not crossing over to the left, small bowel loops in the right half of the abdomen, lack of visualisation of the caecum in the right lower abdomen (Figure 1), and reversed superior mesenteric artery and vein in relation with the vein lying to the left of the artery (Figure 2). A barium contrast study was done and revealed the DJ flexure was limited to the right side of the abdomen, along with the small bowel loops (Figure 3). 

**Figure 4:**
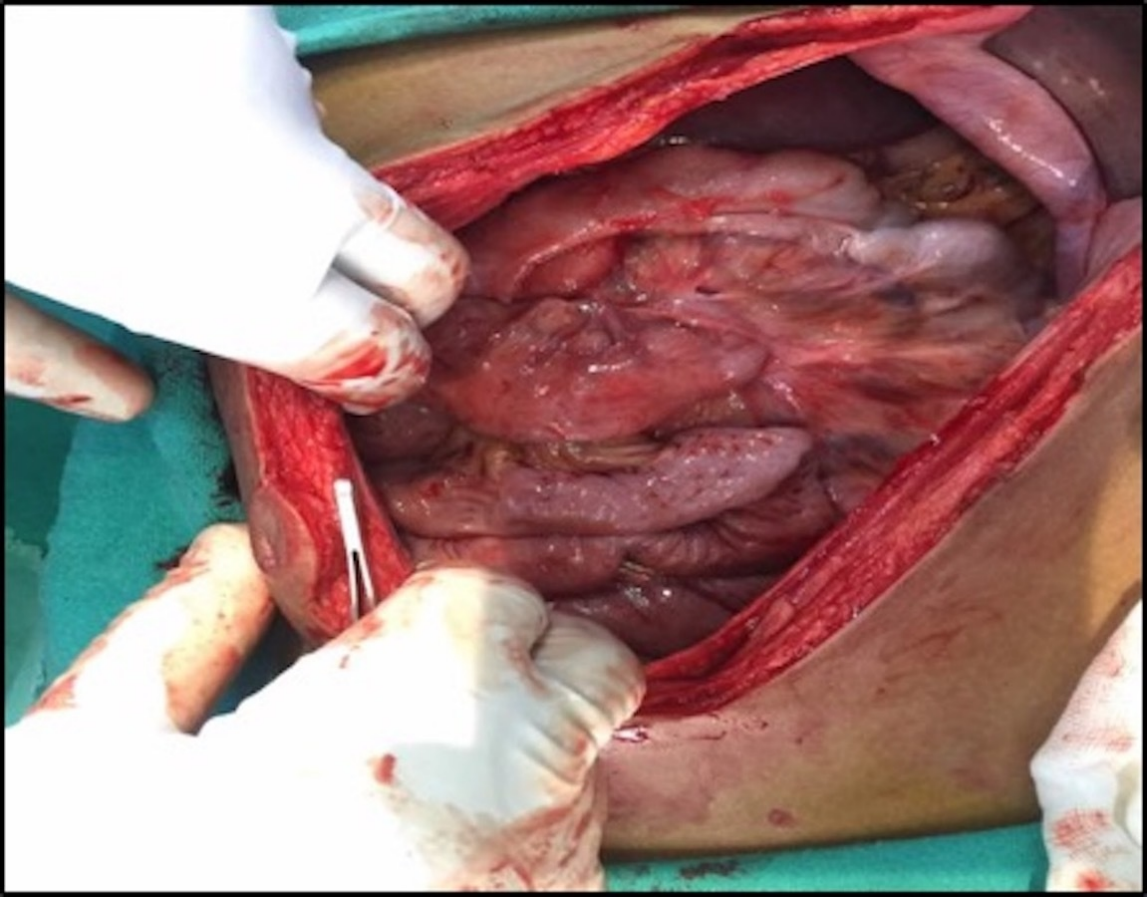
Small bowel loops on the right hiding the colon on opening the abdomen

**Figure 5:**
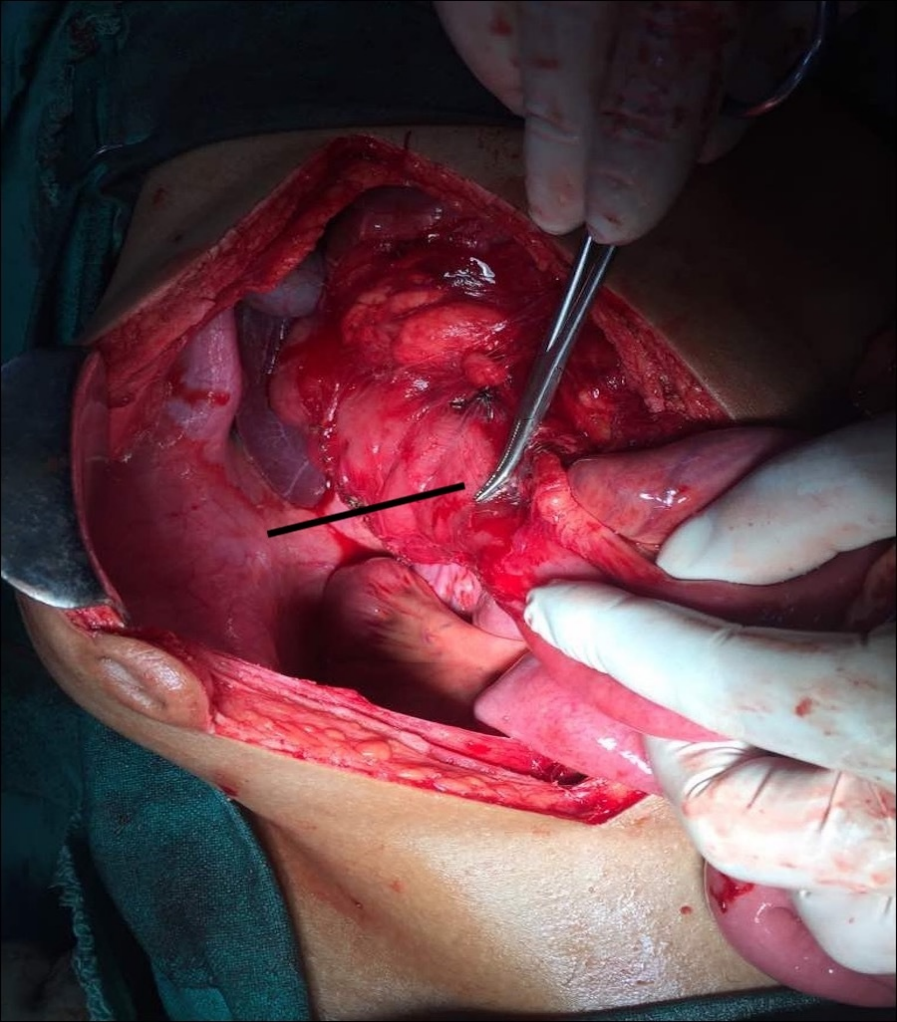
Division of the Ladd's band

A diagnosis of malrotation of the gut was made, and the patient was planned for urgent surgery. Typical findings of malrotation were seen intraoperatively (Figures 4, 5): small bowel loops predominantly on the right side of the abdomen, hiding the colon, and the DJ flexure not crossing the midline and remaining to the right of the midline. Ladd’s procedure was performed. The postoperative period was uneventful and the patient was discharged on day 7. The patient has been doing well for the last three months and is totally symptom-free.

## Discussion

Malrotation of the gut is the complete or partial failure of 270° of counterclockwise rotation of the midgut around the superior mesenteric pedicle. The rotation of the intestines in the embryonic period occurs in three stages [3]:

• Stage 1: Occurs between five to 10 weeks. This involves herniation of the midgut into the umbilical cord, 90-degree counterclockwise rotation and return into the fetal abdomen. • Stage 2: Occurs in week 11 and involves further rotation till 270° in the abdominal cavity. • Stage 3: Involves fixing of the mesentery.

Malrotation in adults is rare and occurs with obscured clinical symptoms, such as recurrent abdominal pain and vomiting, often resulting in multiple hospital visits and posing a diagnostic dilemma to the unpolished surgeon [4-5]. This condition may also present in an acute way, due to midgut volvulus, and may result in intestinal ischaemia and gangrene. The consequences of this are disastrous and often result in massive bowel gangrene, death, and short bowel syndrome if the patient survives. Timely recognition of the condition is the key to survival [5]. A high index of clinical suspicion is necessary, especially in patients with recurrent episodes of abdominal pain and bilious vomiting, no previous surgical history, and no evidence of tuberculosis. Plain abdominal radiographs are not useful and the investigation of choice in adults remains a contrast-enhanced CT scan [5]. The typical findings are reversed relation of superior mesenteric artery (SMA) and superior mesenteric vein (SMV), a whirled appearance of the vasculature entering the volvulus (whirlpool sign), small bowel loops in the right upper abdomen, a lack of visualization of the caecum in the right iliac fossa, dilatation of various duodenal loops, and duodenojejunal flexure to the right (corkscrew sign). The typical reversed relationship of superior mesenteric vessels can be seen on ultrasonography as well. All patients, regardless of age, should undergo surgery as it is impossible to predict the development of catastrophic complications [5]. The procedure of choice is the Ladd’s procedure, be it in elective or emergency settings. This procedure can be performed by a laparoscopic approach as well [5]. This procedure consists of the following steps: delivery of the small bowel and untwisting it counter-clockwise, placing the caecum in the right paravertebral gutter with the bands clearly visible which are divided, kocherization and the widening of the small bowel mesentery, and finally, concluding with an appendectomy. This procedure places the small gut and duodenum on the right side and the large gut on the left. In situations where gangrene has developed, resection of the bowel becomes mandatory.

## Conclusions

A high index of suspicion is needed to diagnose malrotation of the gut in adults. This condition should be suspected in patients with recurrent episodes of abdominal pain and bilious vomiting with no history suggestive of tuberculosis or any history to support an adhesive cause. In the emergency setting, an ultrasound looking for the reversed relation of superior mesenteric vessels can be very useful. Timely diagnosis will prevent the deadly complications of this disease.

## References

[1] Sahu SK, Raghuvanshi S, Sinha A, Sachan PK (2012). Adult intestinal malrotation presenting as midgut volvulus: case report. J Surg Arts.

[2] Kotze PG, Martins JF, Rocha JG (2011). Ladd procedure for adult intestinal malrotation: case report. [Article in Portuguese]. Arq Bras Cir Dig.

[3] Gamblin TC, Stephens RE Jr, Johnson RK, Rothwell M (2003). Adult malrotation: a case report and review of the literature. Curr Surg.

[4] Emanuwa OF, Ayantunde AA, Davies TW (2011). Midgut malrotation first presenting as acute bowel obstruction in adulthood: a case report and literature review. World J Emerg Surg.

[5] Zengin A, Uçar Bİ, Düzgün ŞA (2016). Adult midgut malrotation presented with acute bowel obstruction and ischemia. Int J Surg Case Rep.

